# Enzyme-Mediated Lignocellulose Liquefaction Is Highly Substrate-Specific and Influenced by the Substrate Concentration or Rheological Regime

**DOI:** 10.3389/fbioe.2020.00917

**Published:** 2020-08-06

**Authors:** Timo van der Zwan, Alexander Sigg, Jinguang Hu, Richard P. Chandra, Jack N. Saddler

**Affiliations:** ^1^Forest Products Biotechnology and Bioenergy Group, Department of Wood Science, Faculty of Forestry, The University of British Columbia, Vancouver, BC, Canada; ^2^Department of Chemistry, Technical University of Munich, Munich, Germany

**Keywords:** lignocellulose, enzymatic liquefaction, high solids, rheology, enzymatic hydrolysis, viscosity, yield stress

## Abstract

The high viscosities/yield stresses of lignocellulose slurries makes their industrial processing a significant challenge. However, little is known regarding the degree to which liquefaction and its enzymatic requirements are specific to a substrate’s physicochemical and rheological properties. In the work reported here, the substrate- and rheological regime-specificities of liquefaction of various substrates were assessed using real-time in-rheometer viscometry and offline oscillatory rheometry when hydrolyzed by combinations of cellobiohydrolase (*Trichoderma reesei* Cel7A), endoglucanase (*Humicola insolens* Cel45A), glycoside hydrolase (GH) family 10 xylanase, and GH family 11 xylanase. In contrast to previous work that has suggested that endoglucanase activity dominates enzymatic liquefaction, all of the enzymes were shown to have at least some liquefaction capacity depending on the substrate and reaction conditions. The contribution of individual enzymes was found to be influenced by the rheological regime; in the concentrated regime, the cellobiohydrolase outperformed the endoglucanase, achieving 2.4-fold higher yield stress reduction over the same timeframe, whereas the endoglucanase performed best in the semi-dilute regime. It was apparent that the significant differences in rheology and liquefaction mechanisms made it difficult to predict the liquefaction capacity of an enzyme or enzyme cocktail at different substrate concentrations.

## Introduction

An enduring obstacle in the sustainable production of renewable chemicals, fuels, and materials from lignocellulose via enzymatic deconstruction is the challenging rheology of these substrates when used at high concentrations (Nguyen et al., 2015). The use of high substrate concentrations is motivated by the potential to improve process economics through increased volumetric productivity and a reduction in associated process costs (Koppram et al., 2014). However, mixing these concentrated slurries is challenging or even prohibitively expensive (Zhang et al., 2010). Typically, mass and heat transfer issues limit the upper solids loading boundary for bioconversion processes (Hodge et al., 2008), requiring that a balance is struck between substrate concentration and slurry processability.

It has been shown that, during enzymatic hydrolysis, the viscosity or yield stress of lignocellulose slurries decreases in a process termed “liquefaction,” which occurs through various mechanisms including material dilution, particle fragmentation and modification of interparticle interactions (Roche et al., 2009; Thygesen et al., 2014; van der Zwan et al., 2017). To deal with the high viscosities/yield stresses of concentrated lignocellulose slurries, a separate enzymatic liquefaction stage similar to that used in starch-based biorefineries appears promising (Szijártó et al., 2011a), reducing viscosity/yield stress prior to further saccharification. However, it remains unclear to what degree liquefaction is substrate-specific or how the rheological conditions of a given slurry impact the mechanisms and the enzymatic requirements of liquefaction.

The paradigmatic view of cellulose hydrolysis by fungal, non-complexed enzyme systems is that cellobiohydrolases act as the primary catalysts, with additional support from endoglucanases and beta-glucosidases. This primary function of cellobiohydrolases is reflected in the secretomes of fungi such as *Trichoderma reesei*, where cellobiohydrolases comprise up to 80% of the total secreted protein (Herpoël-Gimbert et al., 2008), while endoglucanases make up a relatively small portion (He et al., 2014). However, previous work examining viscosity reduction during enzymatic hydrolysis indicated that endoglucanases played a central role in enzyme-mediated liquefaction (Szijártó et al., 2011a,b; Skovgaard et al., 2014). While endoglucanases are thought to be the dominant viscosity-reducing enzymes because of their ability to catalyze particle fragmentation, cellobiohydrolases have been shown to synergize with endoglucanases to promote fragmentation of cellulose (Walker et al., 1992). Cellobiohydrolases have also been reported to individually fragment cellulose crystals (Jeoh et al., 2013) and hardwood pulp fibers (Suchy et al., 2009). Additionally, the progressive cleavage of cellulose at particle surfaces by cellobiohydrolases could have a “smoothing” effect that reduces interparticle friction in the slurry (Lee et al., 2000; Ander et al., 2008) and reducing fiber entanglement (Kerekes, 2006). Skovgaard et al. (2014) also showed that the combination of xylanase and endoglucanase or a cellulase mixture resulted in more rapid viscosity reduction as well as increased particle fragmentation. Here, it was suggested that xylanases can cause liquefaction directly through solubilization of xylan resulting in material dilution, as well as indirectly aiding liquefaction by increasing substrate accessibility for other enzymes. This was in contrast to the work by Szijártó et al., who found that viscosity reduction was less effective upon partial replacement of endoglucanase with xylanase (Szijártó et al., 2011b). While both studies were conducted using hydrothermally pretreated wheat straw prepared under similar conditions, differences in hemicellulose content (10.8% xylan compared to 3.6%) may have accounted for the different enzyme requirements. In related work, various lignocellulosic materials displayed distinct rheological behavior over the course of enzymatic hydrolysis depending on the solids loading of the slurry (Kadić et al., 2014). Together, this suggests that, like cellulose saccharification (Gao et al., 2011; Hu et al., 2011), enzymatic liquefaction and the contribution of individual enzymes to viscosity reduction is substrate-specific.

The work reported here assessed if enzyme-mediated liquefaction is influenced by the physicochemical properties of a lignocellulosic substrate, if substrate characteristics influenced the enzyme activities required for liquefaction, and the role that the prevailing rheological regime, as defined by the substrate concentration, plays in determining liquefaction behavior. As described in detail below, a hardwood (aspen) was differentially pretreated via acidic steam pretreatment, neutralized steam pretreatment, mechanical pretreatment and delignification, to produce a range of substrates with varying substrate compositions and characteristics. The substrates were subsequently rheologically characterized using rotating vane rheometry (Barnes and Nguyen, 2001; Aït-Kadi et al., 2002), followed by real-time monitoring of enzymatic liquefaction using purified enzymes and both online and offline rheometry assessment of liquefaction dynamics.

## Materials and Methods

### Substrate Pretreatment

Aspen poplar (*Populus tremuloides*) chips with a moisture content of ∼7% were obtained from Alberta-Pacific Forest Industries Inc. (Boyle, AB). Steam pretreatment was performed on 200 g (dry matter) chips in a two-liter reaction vessel with 3% (by mass) SO_2_ as a catalyst at 200°C for 5 min before decompression into a collection vessel. Multiple rounds of pretreated substrate were collected and mixed. The resulting pulp was washed extensively with tap water to eliminate the influence of solubilized components that could interfere with enzymatic catalysis, then vacuum-filtered to a moisture content of ∼70%. This provided the steam-pretreated (SP) substrate.

Neutralized steam pretreatment involved soaking aspen wood chips (200 g dry matter) in sodium bicarbonate (8% by mass) dissolved in 200 ml water ahead of steam pretreatment to neutralize the organic acids liberated from the biomass during pretreatment. The chips were steamed at 210°C for 5 min prior to decompression into a collection vessel, with a final pH of ∼7. The neutrally pretreated substrate was ground using a single pass through a twin-gear juicer (Super Angel, Tustin, CA) to reduce the average particle size. The substrate was then extensively washed with tap water and vacuum-filtered to a moisture content of ∼70%, resulting in the N-SP substrate.

A refiner mechanical pulp (RMP) was made from aspen wood chips by feeding chips through a laboratory disc refiner (Sprout-Waldron) with D2A507 plates (Chandra et al., 2016). The wood chips were refined in three steps with the disc clearance set at a decreasing order from 0.5 to 0.2 mm, and then to 0.1 mm.

Delignification of each of the aspen poplar substrate variants was achieved through an acid chlorite bleaching method (PAPTAC, 2005). N-SP was taken through two rounds of chlorite bleaching to provide the delignified variant, while the SP and RMP substrates were subjected to one.

### Substrate Analysis

The composition of the various substrates was determined using the National Renewable Energy Laboratory standard laboratory analytical protocols (Sluiter et al., 2012). Number-based particle lengths and widths were measured optically using a HiRes Fiber Quality Analyzer (OpTest Equipment Inc., Hawkesbury, ON). The reported particle lengths are the length-weighted mean lengths, used given their correlation to pulp suspension rheology (Derakhshandeh et al., 2011) and their use in the crowding number (Kerekes and Schell, 1995), and represent a dilute sample of 20k fibers measured at 20–40 events per second. Volume-based fiber dimensions were determined using a laser diffraction particle size analyzer (Mastersizer 2000 with Hydro 2000G wet sample dispersion unit; Malvern, Westborough, MA). Samples of each pulp were sonicated while stirred in the dispersion unit until thoroughly suspended, as determined by the diminishing change in laser obscuration and mean particle sizes. Reported values and distributions represent an average of two technical replicates using a refractive index of 1.56. Reported mean particle diameters are the volume moment mean [De Brouckere mean diameter; *D*(4,3)].

The water retention capacity of the substrates was measured on substrate test pads formed in 15-ml centrifuge tubes capped with 325-mesh screens in triplicate, tested according to TAPPI Universal Method 256 (TAPPI, 2011).

### Oscillatory Rheometry

Oscillatory rheometry was performed using an MCR 502 rheometer (Anton-Paar GmbH, Austria; maximum torque 230 mNm, minimum 1 nNm rotation, 0.5 nNm oscillation; maximum angular velocity 314 rad s^–1^, minimum 10^–9^ rad s^–1^) with a four-bladed vane geometry (24.4 mm diameter, with each vane having a height of 19 mm) in a cylindrical cup (28.5 mm diameter, 67 mm height) (Supplementary Figure S6). The cup was filled with 40 ml substrate slurry, thoroughly vortexed in a 50-ml centrifuge tube to ensure homogeneity ahead of addition, and the measuring geometry was lowered halfway into the slurry. Following insertion, the geometry was raised by 1 mm to relieve the normal forces incurred by compression of the slurry particles. For each sample, frequency and stress sweeps were conducted in sequence, for a minimum of three replicates. Frequency sweeps were performed on a logarithmic ramp with thirty 10 s measurement points from 100–1 Hz at a stress amplitude of 20 Pa. Stress sweeps were conducted at a frequency of 10 Hz on a logarithmic ramp, generally from 0.1–100 Pa (depending on the slurry’s linear viscoelastic region) to a value just above the yield stress, as determined by an initial stress sweep. 50 measurement points were taken with a duration of 5 s. The storage modulus (*G*′), loss modulus (*G*″), and complex viscosity (η*^∗^*, the frequency-dependent viscosity with both viscous, *G″*, and elastic, *G*′, contributions, representing the response of the slurry to the shear forces) were recorded as determined by the RheoPlus software (v. 3.61, Anton-Paar). Oscillatory yield stress was determined as the crossover point of the storage and loss moduli curves in the stress sweep (Knutsen and Liberatore, 2009), taken as an average of three replicates ± standard error.

### Enzymes and Their Purification

Protein purification was carried out using a fast protein liquid chromatography system (ÄKTAprime plus, GE Healthcare). *Tr*Cel7A was sequentially purified from the *Trichoderma reesei* cellulase preparation as previously described (Hu et al., 2013).

A crude preparation of *Humicola insolens* Cel45A (*Hi*Cel45A) cloned and expressed in *Aspergillus oryzae* was provided by Novozymes (Davis, CA). The preparation was first desalted (HiPrep 26/10 Desalting Column, GE Healthcare) into 20 mM triethanolamine buffer, pH 7 (buffer A), followed by anion exchange chromatography (Q Sepharose High Performance, GE Healthcare) in a linear gradient from buffer A to buffer B (20 mM triethanolamine, pH 7 with 1 M NaCl).

The glycoside hydrolase family 10 xylanase (GH10Xyn) and family 11 xylanase (GH11Xyn) were purified from commercial xylanase preparations as previously described (Hu et al., 2013).

The collected fractions were concentrated by ultrafiltration in a centrifuge at 4000 × *g* with centrifugal filter units having a 10 kDa nominal molecular weight limit (Amicon Ultra-15, Millipore). The purity and identity of the enzymes was confirmed by sodium dodecyl sulfate–polyacrylamide gel electrophoresis and tandem mass spectrometry as described previously (Pribowo et al., 2012). The commercial enzyme preparation Cellic CTec3 was used directly without purification (kindly provided by Novozymes, Franklinton, NC), having an activity of 196 FPU ml^–1^ and a protein content of 183 mg ml^–1^ (van der Zwan et al., 2019). The concentration of purified *Tr*Cel7A was determined through absorbance at 280 nm measured in a spectrophotometer using an attenuation coefficient of 84.4 mM^–1^ cm^–1^ (Velleste et al., 2010). Concentrations of other purified proteins was determined using a microplate-based bicinchoninic acid assay according to the kit instructions (Pierce BCA Protein Assay Kit; Thermo Fisher Scientific).

### Enzyme Activity Assays

The cellobiohydrolase activity was assessed in microplate assays using the chromogenic substrate *p*-nitrophenyl-β-D-cellobioside in 100 μl reactions with 50 mM sodium acetate buffer pH 5 at 50°C, with the addition of 5 mM gluconolactone. Stop solution (100 μl, 1 M glycine/0.8 M NaOH) was added at 10, 20, and 30 min to five replicates each and the color developed as a result of *p*-nitrophenol liberation was measured at 405 nm on a microplate spectrophotometer. The amount of liberated *p*-nitrophenol was calculated against a standard curve of *p*-nitrophenol dilutions.

Endoglucanase and xylanase activities were respectively assessed using 0.7% (by mass) solutions of carboxymethyl cellulose (90 kDa average molecular weight, 0.7 carboxymethyl groups per anhydroglucose unit; Sigma) and birchwood xylan (Sigma) in 100 μl reactions with 50 mM sodium acetate buffer pH 5 on low-evaporation microplates at 50°C. Appropriately diluted enzyme samples were incubated with substrates for 10, 20, and 30 min (five replicates each) and stopped by the addition of a dinitrosalicylic acid (DNS) reagent solution, prepared as previously described (Adney and Baker, 2008). The microplates were incubated at 105°C for 30 min to allow reaction of DNS with liberated saccharides and the color produced was measured at 540 nm on a microplate spectrophotometer following cooling to room temperature. A standard curve was prepared with serial dilutions of glucose and xylose treated in the same manner with DNS.

Measured enzyme activities are summarized in Supplementary Table S1.

### Enzymatic Hydrolysis

For in-rheometer real-time monitoring of liquefaction, enzymatic hydrolysis was conducted in a 20-ml 2.5% (m/m) solids slurry made up with 50 mM sodium acetate buffer, pH 5, at 50°C loaded into a cylindrical cup in the rheometer described above. Temperature control was achieved using a C-PTD 200 Peltier device (Anton-Paar GmbH, Austria) in combination with counter-cooling using an external water circulator. Prior to enzyme addition, the slurry was warmed to 50°C under constant stirring at a shear rate of 60 s^–1^ for 5 min and was confirmed to maintain the same viscosity as the no-enzyme control. The measuring geometry was then raised out of the slurry and the enzyme was added and thoroughly mixed into the slurry with a spatula. The geometry was then immediately lowered back into the slurry and shear initiated. Reactions were run at a constant shear rate of 60 s^–1^ for 1 h, measuring viscosity every 10 s. Laboratory film stretched around a cover with a narrow cutout for the measurement geometry was used to minimize evaporation.

The offline measurement of liquefaction of 12.5% (m/m) solids slurries was conducted in 50 mM sodium acetate buffer, pH 5 in 100-ml screw-cap bottles rotated at 10 RPM on a rotator drive unit (STR4, Stuart, Staffordshire, United Kingdom) within an incubator maintained at 50°C. Experiments were done in duplicate. The reactions were stopped through heat-inactivation in a water bath at 95°C for 30 min and the slurries stored at 4°C until further analysis.

Sugar release was measured against a standard curve using high performance anion exchange chromatography with pulsed amperometric detection (HPAEC-PAD) with fucose as an internal standard, as described previously (Burkhardt et al., 2013). Twenty microliter of appropriately diluted hydrolysate was loaded onto an anion exchange column (CarboPac PA1; Dionex, Sunnyvale, CA) on a high-performance liquid chromatography system (DX-3000, Dionex) using deionized water as column eluent at 1 ml min^–1^. 0.2 M NaOH was mixed in post-column ahead of the gold electrode electrochemical detector at 0.5 ml min^–1^. The column was reconditioned using 1 M NaOH after each run. Cellulose (glucan) hydrolysis extent was calculated as the amount of glucose produced over the potential glucose released from the substrate.

### Statistical Analysis

Unless otherwise noted, data is reported as the mean ± standard error. Where appropriate, statistical significance was tested using analysis of variance followed by Tukey *post hoc* tests (*α* = 0.05).

## Results

### Chemical, Physical and Rheological Characterization of the Pretreated Substrates

To assess the potential substrate-dependency of liquefaction dynamics, a hardwood (aspen) was differentially pretreated to produce a range of substrates varying in composition and physicochemical properties (Table 1). The pretreatments included acidic steam pretreatment (SP), neutralized steam pretreatment (with pH neutralization to avoid acid hydrolysis of hemicellulose during the pretreatment; N-SP), and mechanical refining (refiner mechanical pulp; RMP). In addition, a partially delignified variant (-Delig) was produced from each of the substrates. It was apparent that hemicellulose was substantially removed under the acidic conditions of SP, while being retained in the N-SP and RMP substrates (Table 1). Acidic steam pretreatment also resulted in more particle fragmentation, as indicated by particle width, length, and diameter, as well as the particle size distributions (Supplementary Figure S1). Despite their differing pretreatment histories, the SP, N-SP, and RMP substrates all showed similar water retention values, which increased substantially following partial delignification (Table 1). All of the substrates were readily hydrolyzed by a commercial enzyme preparation, with the exception of the non-delignified mechanically refined RMP substrate (Supplementary Figure S2).

**TABLE 1:** Composition and properties of the pretreated aspen substrates.

**Substrate**	**Composition (% dry matter)**				**Water retention (g⋅g^–1^)**	**Particle width (μm)**	**Particle length^a^ (μm)**	**Particle diameter^b^ (μm)**
	**Glucan**	**Lignin**	**Xylan**	**Mannan**				
SP	60 (0.2)	32 (1.5)	1.6 (0.1)	3.0 (0.1)	2.12 (0.02)	21.9 (0.5)	447 (4.2)	116 (2.4)
SP-Delig	91 (1.5)	4.5 (0.5)	1.9 (0.1)	2.9 (0.1)	2.88 (0.01)	21.3 (0.5)	461 (5.0)	114 (1.0)
N-SP	58 (0.1)	30 (0.6)	11 (0.1)	3.0 (0.1)	2.20 (0.08)	23.4 (0.3)	744 (6.1)	208 (3.7)
N-SP-Delig	85 (0.5)	4.3 (1.4)	12 (0.1)	2.9 (0.1)	2.92 (0.03)	21.4 (0.2)	706 (6.8)	206 (0.2)
RMP	45 (0.3)	29 (0.3)	13 (0.1)	4.8 (0.1)	2.02 (0.01)	25.5 (0.7)	526 (5.3)	216 (2.7)
RMP-Delig	63 (0.2)	19 (0.4)	14 (0.2)	4.8 (0.2)	2.71 (0.09)	27.3 (0.8)	488 (5.2)	235 (3.4)

**^*a*^Length-weighted mean obtained with optical analysis. ^*b*^Volume-weighted mean obtained with laser diffraction analysis. Bracketed values indicate standard error of the mean.**

**FIGURE 1:**
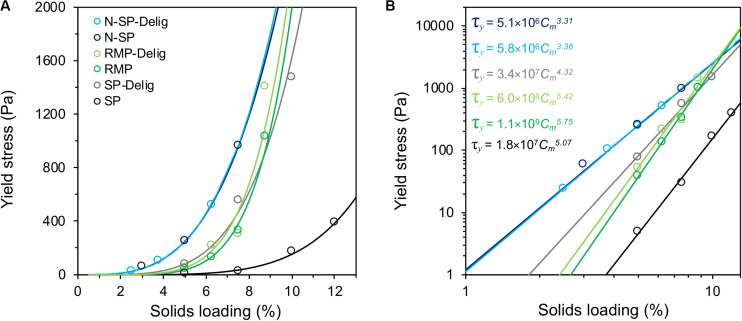
Slurry yield stress of pretreated aspen substrate variants at different solids loadings, with power-law regression fits shown on linear **(A)** and log–log plots **(B)**. The power law relationships of the substrates are listed in **(B)**, calculated using the fractional consistency, *C_m_*, in the form *τ_*y*_* = *aC_*m*_^*b*^*.

The substrates demonstrated widely varying rheological behavior with the acidic steam-pretreated SP substrate showing, by far, the lowest yield stress (Figure 1A). The yield stress of SP slurries was at least an order of magnitude lower than any other substrate (Figure 1B). However, following partial delignification, the yield stress of the SP substrate increased by about an order of magnitude. In contrast, a marginal increase in the yield stress of the RMP substrate was observed following delignification, while delignification of the N-SP substrate appeared to have no influence.

### In-Rheometer Liquefaction Baseline Using a CTec3 Cellulase Preparation

**FIGURE 2:**
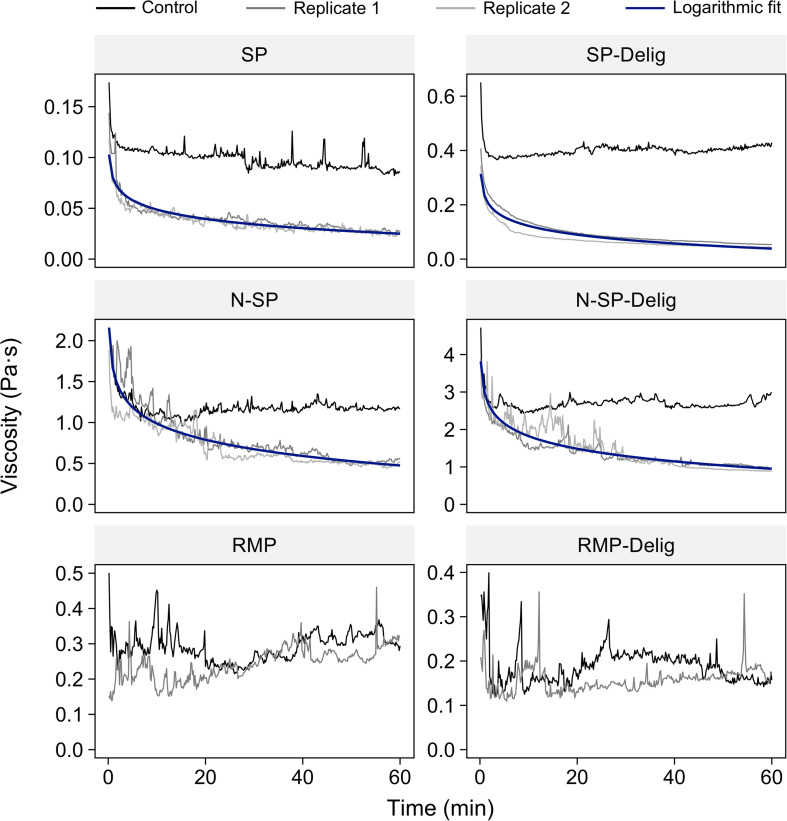
In-rheometer assessment of pretreated aspen substrates hydrolyzed by Cellic CTec3 at 20 mg_protein_ g_cellulose_^–1^. For visual clarity, only one replicate is shown for the RMP and RMP-Delig substrates.

Prior to assessing possible enzyme-mediated liquefaction using purified enzymes, a baseline for real-time in-rheometer liquefaction was established after hydrolysis using a commercial cellulase preparation (CTec 3; Figure 2) known to contain cellobiohydrolases, endoglucanases, xylanases and various accessory enzymes. Although considerable viscosity reduction was evident with the acidic steam-pretreated SP and neutralized steam-pretreated N-SP substrates and their partially delignified variants, no viscosity reduction was detected with the mechanically refined RMP substrates. Accordingly, the rest of the work was limited to the acid and neutral steam-pretreated substrates and their delignified variants.

### Assessment of In-Rheometer Liquefaction Using Purified Enzymes

**FIGURE 3:**
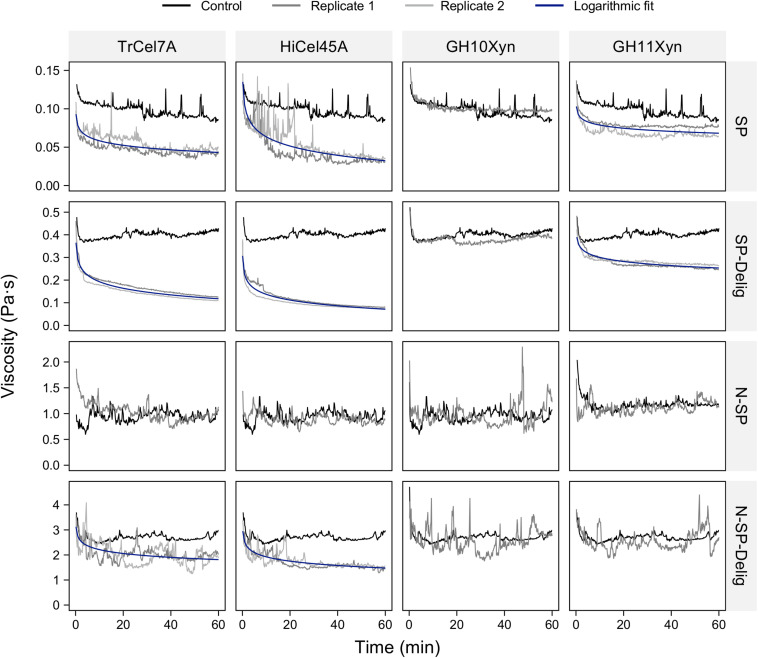
In-rheometer assessment of the pretreated aspen substrates (as labeled in rows) at 2.5% (m/m) solids after addition of individual purified enzymes (as labeled in columns) at 20 mg_protein_ g_cellulose_^–1^. For visual clarity, only one replicate is shown for those reactions where no viscosity reduction was observed.

Possible in-rheometer liquefaction of the SP and N-SP substrates and their delignified variants was assessed using purified cellobiohydrolase (*Tr*Cel7A), endoglucanase (*Hi*Cel45A), glycoside hydrolase family 10 xylanase (GH10Xyn) and glycoside hydrolase family 11 xylanase (GH11Xyn) (Figure 3). The addition of cellobiohydrolase *Tr*Cel7A and endoglucanase *Hi*Cel45A each resulted in substantial viscosity reduction when added to the SP, SP-Delig, and N-SP-Delig substrates. In each case, the addition of endoglucanase resulted in a slightly lower viscosity endpoint than cellobiohydrolase addition. In contrast, no viscosity reduction was evident when either the endoglucanase or the cellobiohydrolase was added to the N-SP substrate. Although the addition of GH11Xyn resulted in some viscosity reduction for the SP and SP-Delig substrates, but not for the N-SP substrate or its delignified variant, the GH10Xyn did not result in viscosity reduction for any of the substrates.

Neither the cellobiohydrolase *Tr*Cel7A nor the endoglucanase *Hi*Cel45A reduced slurry viscosity to the same extent as the CTec3, although the endoglucanase came closest. With the SP substrate, the addition of CTec3 reduced the viscosity to a fractional endpoint of 0.3 (expressed as a fraction of the average control viscosity), in comparison to 0.39 for the endoglucanase-treated slurries and 0.51 for the cellobiohydrolase. For the SP-Delig substrate, the endpoints were 0.1 for the cellulase preparation, 0.17 for the endoglucanase and 0.28 for the cellobiohydrolase. For the N-SP-Delig substrate, the endpoints were 0.32, 0.50, and 0.62, respectively.

While relatively high enzyme concentrations were used to minimize the influence of unproductive adsorption, liquefaction capacity loss was minimal when the enzyme load was reduced to as little as 1.25 mg_protein_ g_cellulose_^–1^ (Supplementary Figure S3).

### Assessment of In-Rheometer Liquefaction Using Enzyme Combinations

**FIGURE 4:**
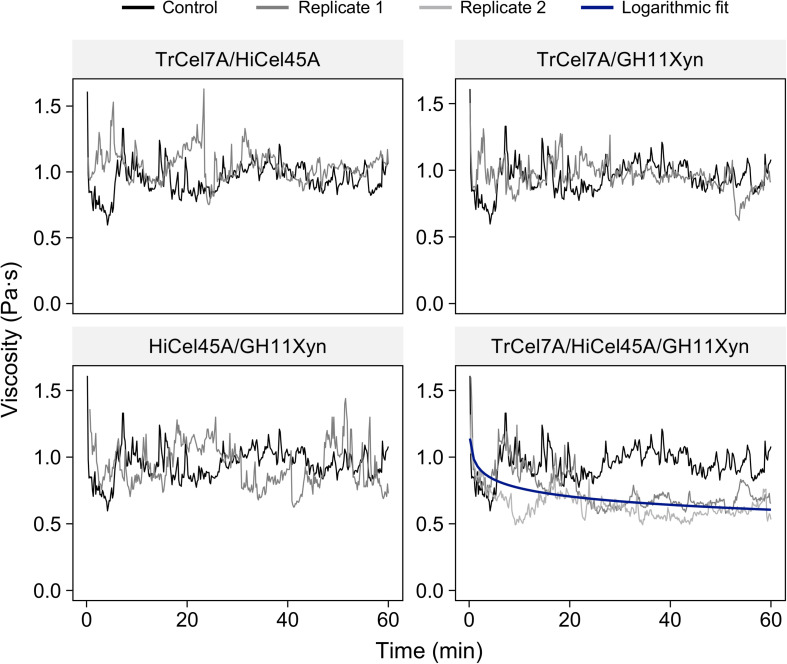
In-rheometer reactions of neutrally steam-pretreated aspen (N-SP) at 2.5% (m/m) with purified enzyme combinations as indicated, at a total of 20 mg_protein_ g_cellulose_^–1^ with equal mass ratios per enzyme. To enhance clarity, only one replicate is shown for reactions where no viscosity reduction was observed.

To try to better elucidate the enzymatic requirements for the liquefaction of the N-SP substrate and the associated implications on the substrate-specificity of enzymatic liquefaction, in-rheometer reactions were conducted with equal-mass-ratio mixes of the purified enzymes (Figure 4). It was apparent that none of the binary combinations of enzymes (*Tr*Cel7A/*Hi*Cel45A, *Tr*Cel7A/GH11Xyn, or *Hi*Cel45A/GH11Xyn) resulted in viscosity reduction, and it was not until the cellobiohydrolase, endoglucanase and xylanase were added in a ternary combination (*Tr*Cel7A, *Hi*Cel45A, and GH11Xyn) that significant reduction in viscosity could be observed, to an endpoint mean viscosity of 0.59 ± 0.06 Pa⋅s compared to 0.96 ± 0.085 Pa⋅s for the control (taken as the mean ± standard deviation of the control in the stabilized region from 20 to 60 min). Viscosity reduction was also observed when the GH10Xyn enzyme was additionally added in a quaternary combination (Supplementary Figure S4), to an endpoint of 0.60 ± 0.009 Pa⋅s.

### Assessment of High-Solids Liquefaction Using Purified Enzymes Measured via Offline Rheometry

**FIGURE 5:**
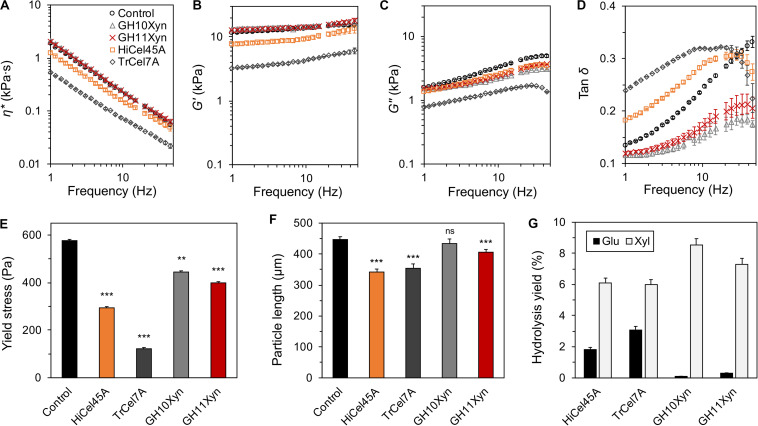
Purified enzyme reactions with steam-pretreated aspen (SP) at 12.5% (m/m) solids with 10 mg_protein_ g_cellulose_^–1^ for 4 h at 50°C. Oscillatory rheometry was used to determine complex viscosity (η*; **A**), storage (G′) and loss (G″) moduli **(B,C)**, tan δ **(D)**, and yield stress **(E)**; mean particle lengths were determined using optical fiber analysis **(F)**; and liberated carbohydrates were measured by HPAEC-PAD following depolymerization of oligomers **(G)**. Error bars indicate standard error of the mean. In **(E,F)**, statistical significance compared to the control is indicated (***p < 0.001; **p < 0.01; ns, not significant).

We next determined whether the enzymatic liquefaction observed at low solids loadings, as measured via in-rheometer real-time monitoring in a semi-dilute regime, was representative of enzyme-mediated liquefaction in a concentrated (high solids loading) regime, where the slurry behaves as a structured fluid or soft solid. When enzymatic hydrolysis of the SP substrate was conducted at a 12.5% (m/m) solids loading, the slurry displayed clear soft-solid behavior with a yield stress of ∼590 Pa (Figure 5). Although just the cellobiohydrolase *Tr*Cel7A-treated and endoglucanase *Hi*Cel45A-treated slurries resulted in a reduction in the complex viscosity (η^∗^; Figure 5A) and storage modulus (*G*′; Figure 5B), all enzyme treatments resulted in a reduction in the loss modulus (*G*″; Figure 5C). For both the cellobiohydrolase and endoglucanase-treated slurries, the reduction in the storage modulus was larger than the reduction in their loss modulus, resulting in a higher loss tangent (tan δ; Figure 5D) than the control. Despite the variable viscoelastic behavior following treatment with the different enzymes, each of the enzymes caused significant yield stress reduction (Figure 5E). This included the GH family 10 xylanase, which based on the earlier semi-dilute regime in-rheometer reactions, did not appear to mediate any slurry liquefaction. It is worth noting that the observed yield stress reduction following treatment with GH10Xyn occurred in the absence of any reduction of particle length, while treatment with each of the other enzymes resulted in significant decreases (Figure 5F). All of the enzymes catalyzed appreciable extents of hydrolysis of the substrates (Figure 5G and Supplementary Figure S5).

## Discussion

It was previously demonstrated (van der Zwan et al., 2017) that enzymatic liquefaction results from the combined action of material dilution, particle fragmentation and alteration of interparticle interactions (Supplementary Figure S7) with the relative contribution of each mechanism dependent on the properties of the substrate and the slurry concentration. Related work that described the relative contribution of different enzymes to liquefaction suggested that cellobiohydrolases contributed little to viscosity reduction (Szijártó et al., 2011a,b). However, in contrast to previous reports, in the work reported here, the addition of cellobiohydrolase *Tr*Cel7A resulted in considerable viscosity reduction, comparable to that resulting after endoglucanase *Hi*Cel45A addition. These results are in agreement with related work that has shown that the addition of cellobiohydrolase *Tr*Cel7A resulted in the fragmentation of cellulose, either in synergy with endoglucanases (Walker et al., 1992) or acting alone (Jeoh et al., 2013).

While enzymatic digestibility of the neutral steam-pretreated and mechanically refined substrates increased substantially following delignification, this was not the case for the acidic steam-pretreated substrate. This apparent reduction in digestibility following delignification could potentially be attributed to a collapse of cell wall ultrastructure and increased interfibril association of cellulose induced by the high degree of lignin removal in the acidic steam-pretreated substrate, causing reduced accessibility of cellulose toward enzymes and thereby reducing substrate digestibility (Ding et al., 2018). At the same time, the high degree of delignification of the acidic steam-pretreated substrate resulted in a substantial increase in its yield stress and viscosity, likely due to the increased water retention capacity of the delignified substrate, and perhaps also due to the increased strength of interparticle interactions through greater hydrogen bonding at cellulose surfaces unobscured by lignin (Shao and Li, 2006). Although the neutral steam-pretreated substrate exhibited a similar degree of delignification, there was no apparent change in slurry yield stress. It is possible that the high hemicellulose content of this substrate prevented the ultrastructural changes and high interparticle interaction seen with the acidic steam-pretreated substrate, with hemicellulose acting as a spacer preventing interfibril association (Pere et al., 2019).

Given the high xylan content of the aspen substrates and the high degree of synergy of xylanases observed with cellulases during enzymatic hydrolysis of xylan-rich substrates (Hu et al., 2011, 2013), it was anticipated that the xylanases used in the present work might contribute to substrate liquefaction. However, only the GH11Xyn enhanced viscosity reduction and only with the acidic steam-pretreated substrates, despite the much higher xylan content of the neutrally steam-pretreated substrates. It was possible that the hemicellulose in the neutrally steam-pretreated substrates restricted enzyme access to the cellulose while becoming exposed following acidic steam pretreatment (Langan et al., 2014). However, it was surprising that the GH10Xyn did not result in any viscosity reduction despite the supposed substrate promiscuity of GH family 10 xylanases (Collins et al., 2005) and its high activity as measured on model substrates (Supplementary Table S1). The difference in liquefaction capacity of the GH10 and GH11 xylanases could potentially be attributed to the distinct substrate specificities of the two xylanase families, with the targeted attack of GH11 xylanases toward the unsubstituted backbone of xylan, as opposed to the generalist activity of GH10 action at substituted and unsubstituted xylose residues (Collins et al., 2005), perhaps being beneficial at the early stages of enzymatic hydrolysis for substrate liquefaction. It may also be that at the low solids loading used for the in-rheometer reactions, particle fragmentation is the primary mechanism of liquefaction affecting slurry viscosity, and given that no particle fragmentation could be detected following treatment with GH10Xyn, its liquefaction capacity could not be ascertained in these conditions. This would likewise explain the lack of viscosity reduction observed here with 60-min in-rheometer reactions with the mechanically refined substrates, which even with a full cellulolytic enzyme cocktail do not display particle size reduction over the course of enzymatic digestion (van der Zwan et al., 2017). For such substrates, material dilution through solubilization of polysaccharides and modification of interparticle interactions through enzyme action at particle surfaces are likely to dominate liquefaction when examined over longer reaction times and at higher substrate concentrations.

The in-rheometer control reactions consistently displayed an initial reduction in viscosity down to their relative equilibrium viscosity within the first 5 min of shear induction. This likely arose due to an initial re-homogenization of the slurry in line with the shear of the vane geometry following enzyme addition and manual mixing of the enzyme with the substrate slurry in the rheometer cup, which was necessary to ensure proper dispersal of the enzyme throughout the slurry.

While none of the purified enzymes either applied individually or in binary combinations resulted in a viscosity reduction of the neutrally steam-pretreated (N-SP) substrate, the cellobiohydrolase, endoglucanase and xylanase combination did result in viscosity reduction. Thus, it was likely that the high xylan content of this substrate required the addition of the xylanase to increase cellulase accessibility to the cellulose.

While being a powerful tool for real-time analysis, in-rheometer monitoring of enzymatic liquefaction of fibrous lignocellulosic materials is inherently limited to the analysis of low-yield-stress/low-viscosity slurries as the mixing geometry must be able to promote continuous movement of the entire slurry without formation of a slip layer that would lead to sample fracture (Stickel et al., 2009). In previous in-rheometer liquefaction studies (Szijártó et al., 2011a, b; Skovgaard et al., 2014; Le et al., 2017) relatively low solids loadings/low-viscosity slurries were therefore used, despite the fact that other studies have demonstrated that enzymatic hydrolysis kinetics differ substantially between low- and high-solids rheological regimes (Knutsen and Liberatore, 2009; Modenbach and Nokes, 2013; Nguyen et al., 2015). Thus, we also examined whether the enzymatic liquefaction observed at low solids loadings, as measured via in-rheometer real-time monitoring in a semi-dilute regime, was representative of enzyme-mediated liquefaction in a concentrated (high solids loading) regime, where the slurry behaves as a structured fluid or soft solid.

Considerable liquefaction differences were observed between high and low substrate concentrations. While *Hi*Cel45A endoglucanase mediated the highest degree of liquefaction with the acidic steam-pretreated substrate at a low solids loading, at a high solids loading, the *Tr*Cel7A cellobiohydrolase resulted in a 2.4-fold lower yield stress endpoint after 4 h compared to the endoglucanase. Although another fungal GH45 endoglucanase was recently shown to outperform GH5 and GH7 endoglucanases at high substrate concentrations in reducing pulp viscosity and degree of polymerization (Rahikainen et al., 2019), the *H. insolens* GH45 endoglucanase used here could not achieve a degree of liquefaction similar to that of the cellobiohydrolase at high substrate concentration. Similarly, when the xylanases were added individually, no viscosity reduction was observed at a low solid loading. However, an up to 30% reduction in yield stress was observed after a 4 h reaction at a high solids loading. It was likely that these differences reflect the degree to which the different liquefaction mechanisms contribute to slurry liquefaction depending on the strength of interparticle interaction and the amount of interstitial water in the slurry, as influenced by the rheological regime.

While in general the extents of hydrolysis with the purified enzymes following 4 h reaction at 12.5% solids were low (at most 3.1% yield of glucose and 8.5% yield of xylose), these small extents of hydrolysis alongside large changes in slurry rheology reflect previous findings in the literature (Skovgaard et al., 2014; Gourlay et al., 2018). Surprisingly, some xylan hydrolysis was evident for all reactions, even for reactions with cellobiohydrolase. It is possible that the xylose that was detected was derived from loosely associated xylose or xylooligosaccharides that had been released following interaction of enzymes with the substrate. Such release of xylose has previously been reported following treatment of pretreated lignocellulose with the non-hydrolytic protein swollenin (Gourlay et al., 2013), and it is possible that cellobiohydrolase here had a similar effect.

Recent work (Du et al., 2020) has also shown that the optimal ratios of endoglucanase to cellobiohydrolase varies for different substrates at high solids loadings. For example, cellobiohydrolase was found to be more influential when hydrolyzing acid-pretreated substrates at high solids loadings while hydrolysis of alkaline-pretreated substrates required a high proportion of endoglucanase. To achieve effective liquefaction, and as supported by other recent work (Weiss et al., 2019), it is likely that enzyme optimization for liquefaction will be dependent on the substrate nature and its initial concentration, as liquefaction efficiency at low solids loadings does not necessarily correlate with the observed efficiency at higher solids loadings.

It was also apparent that, as reported earlier (Skovgaard et al., 2014; Gourlay et al., 2018), the relatively low extent of hydrolysis resulted in large changes in slurry rheology. As significant rheological changes can be obtained using low enzyme loadings, future work might assess if the rate-limiting liquefaction step can be further enhanced by tailoring enzyme cocktails specific to the liquefaction requirements of a substrate, such as with thermostable enzymes during the cool-down phase following biomass pretreatment (Brunecky et al., 2014; Hämäläinen et al., 2016).

## Data Availability Statement

The raw data supporting the conclusions of this article will be made available by the authors, without undue reservation, to any qualified researcher.

## Author Contributions

TZ, RC, JH, and JS designed the study. TZ, RC, and JH conducted substrate pretreatment and analysis. TZ, JH, and AS purified and characterized enzymes. TZ and AS performed rheological experiments and interpreted the data. TZ and JS drafted the manuscript. All authors read and approved the final manuscript.

## Conflict of Interest

The authors declare that the research was conducted in the absence of any commercial or financial relationships that could be construed as a potential conflict of interest.
