# Supplementary material for: Enzyme-Mediated Lignocellulose Liquefaction Is Highly Substrate-Specific and Influenced by the Substrate Concentration or Rheological Regime
Source: Front Bioeng Biotechnol. 2020 Aug 6;8:917. doi: 10.3389/fbioe.2020.00917 (PMC7423843; doi:10.3389/fbioe.2020.00917)
Supplement: Supplementary file 1 [file Data_Sheet_1.docx]

Supplementary Material

**Table S1.** Enzyme activities of the purified enzymes used in the study.

| **Enzyme** | **Substrate** | **Enzyme activity** | **Specific activity** (nkat mg^-1^) |
| --- | --- | --- | --- |
| *Hi*Cel45A | carboxymethyl cellulose | endoglucanase | 74.8 |
|  | *p*-nitrophenyl β-D-cellobioside | cellobiohydrolase | na^a^ |
|  | birchwood xylan | xylanase | 0.13 |
| *Tr*Cel7A | *p*-nitrophenyl β-D-cellobioside | cellobiohydrolase | 6.8 |
|  | carboxymethyl cellulose | endoglucanase | na^b^ |
| GH10Xyn | birchwood xylan | xylanase | 898 |
| GH11Xyn | birchwood xylan | xylanase | 670 |
| ^a^negligible activity; ^b^after concentration through ultrafiltration, the specific activity was found to be in the fkatal mg^-1^ range | | | |

**
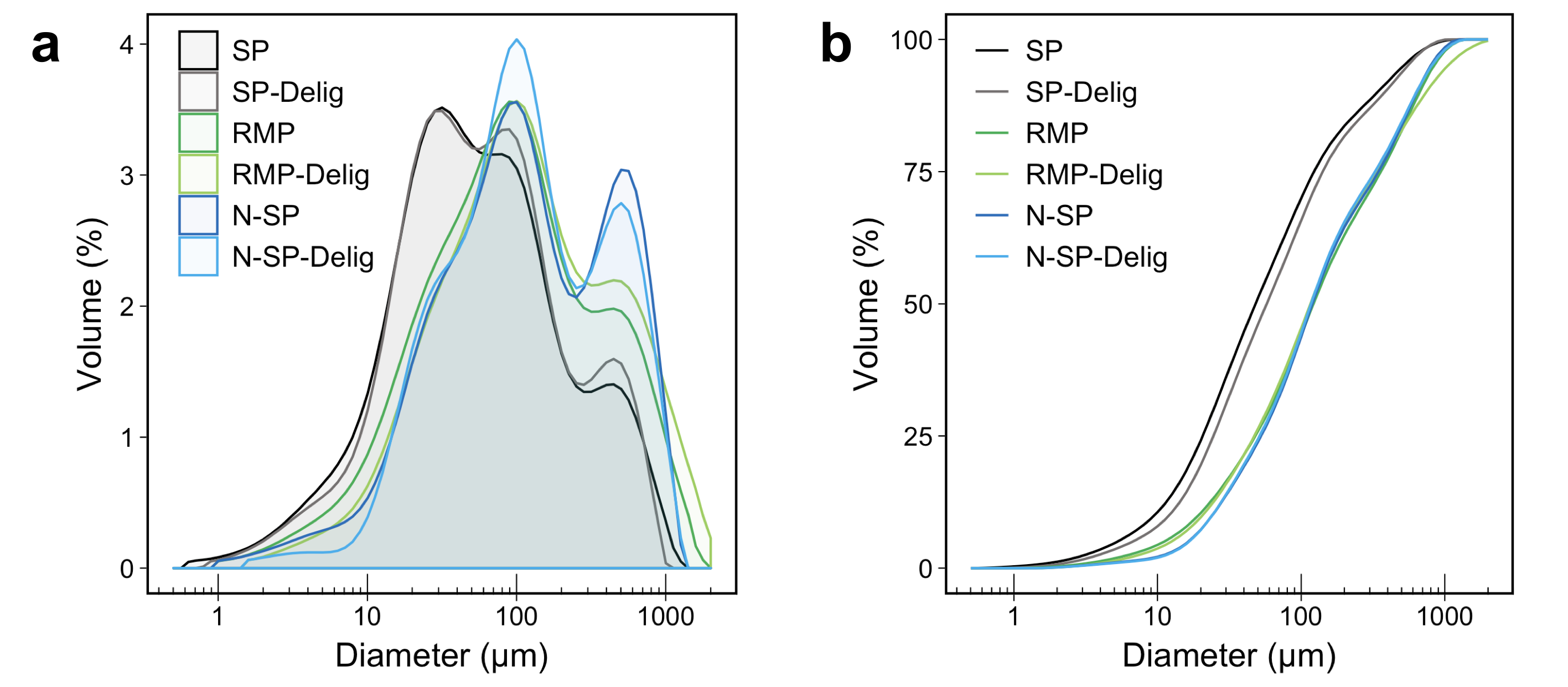
**

**Fig. S1.** Volume-based particle size distributions (**a**) and cumulative distributions (**b**) of the pretreated aspen substrate variants obtained through laser diffraction particle size analysis.

**
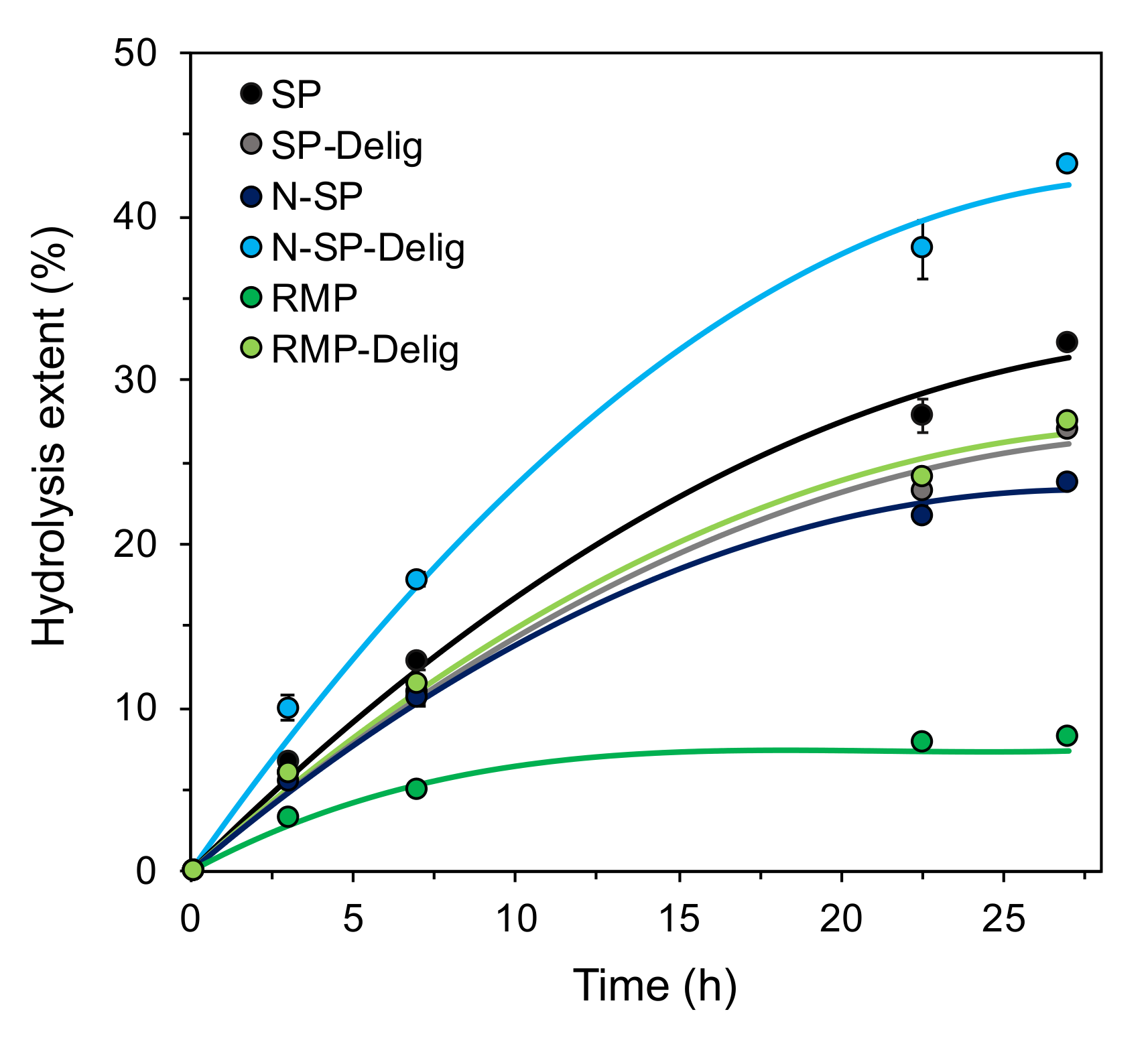
**

**Fig. S2.** Enzymatic hydrolysis of the aspen substrate variants at 2 % (m/m) solids, with 2.5 mg_protein_ g_cellulose_^-1^ of commercial cellulase cocktail (CTec3), in 1-ml reactions in 2-ml tubes, 50 mM sodium acetate buffer, pH 5, 50 °C, rotated at 10 rpm in a hybridization incubator.

**
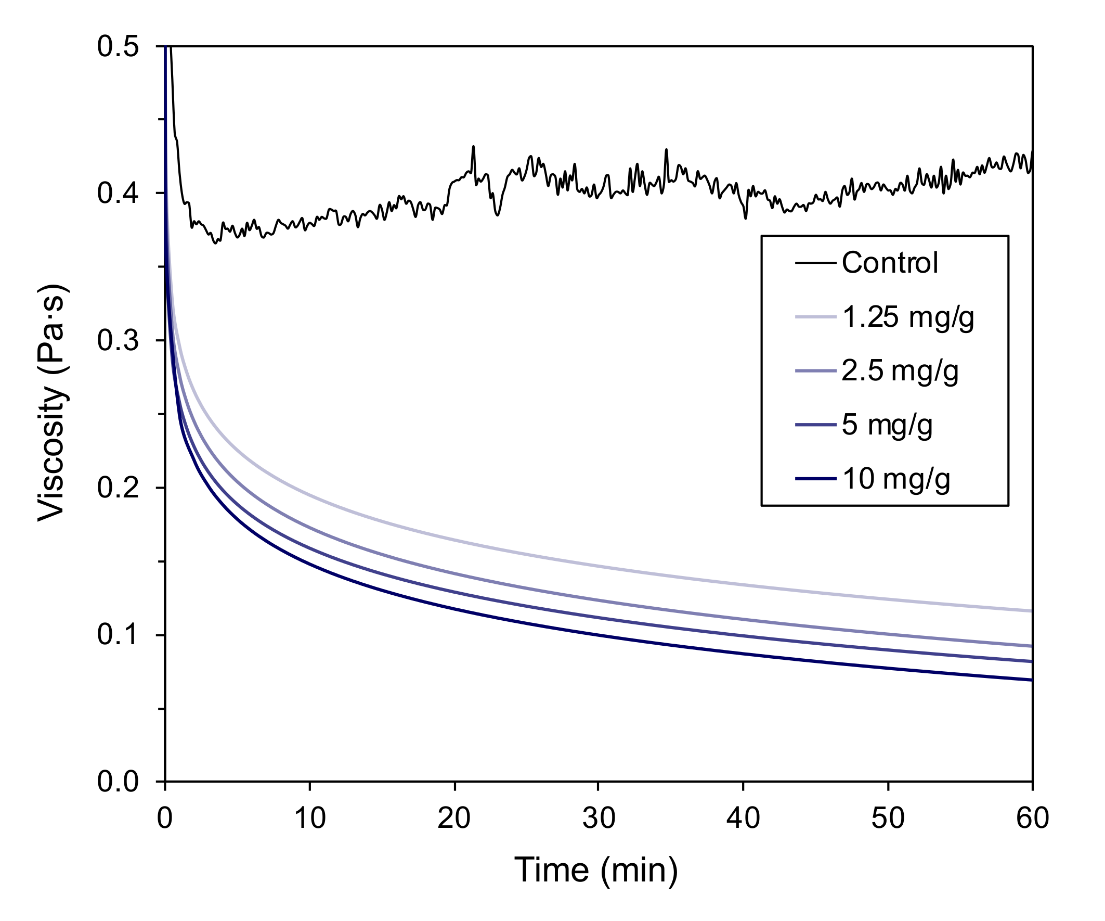
**

Fig. S3. In-rheometer reactions of delignified steam-pretreated aspen (SP-Delig) at 2.5 % (m/m) solids with HiCel45A at varying enzyme loadings, as indicated in mg_protein_ g_cellulose_^-1^. Viscosity values of duplicate reactions were averaged and a logarithmic curve in the form y = a + b⋅ln(x) was fitted to the averaged viscosity data against time for each enzyme loading; individual replicate curves are not shown to enhance clarity.

**
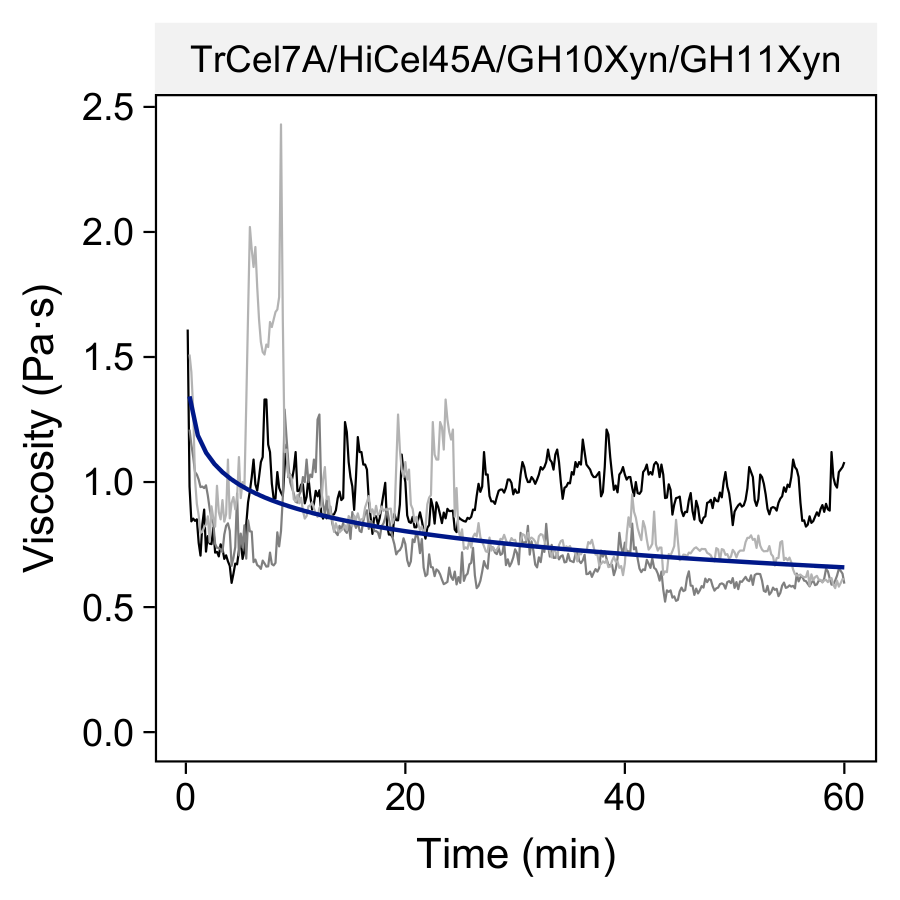
**

**Fig. S4.** In-rheometer reactions of neutrally steam-pretreated aspen poplar (N-SP) at 2.5 % (m/m) solids with *Tr*Cel7A, *Hi*Cel45A, GH10Xyn, and GH11Xyn at a loading of 5 mg_protein_ g_cellulose_^-1^ per enzyme.


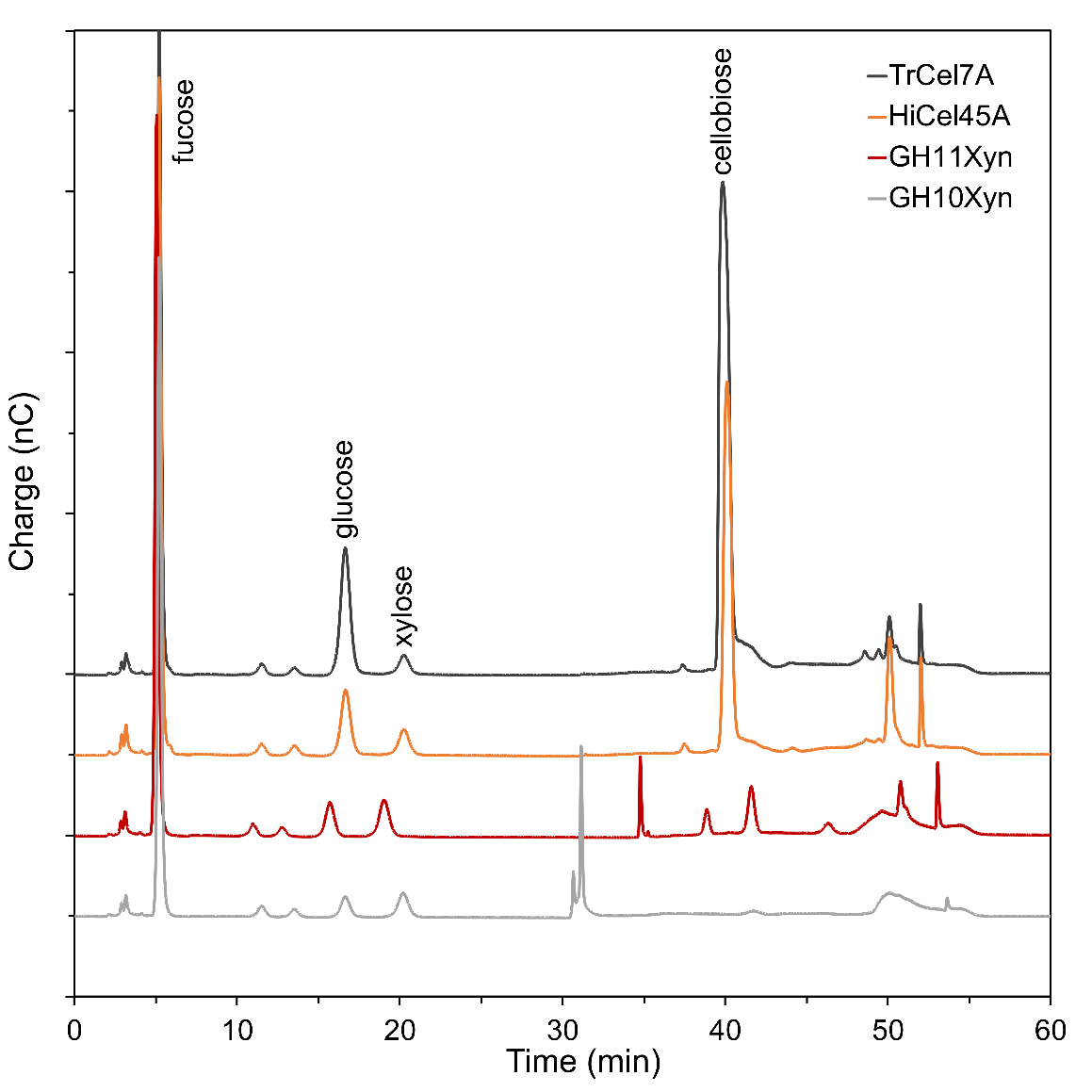


**Fig. S5.** HPAEC-PAD chromatograms of SP hydrolysates after enzymatic hydrolysis at 10 % (m/m) solids for 4 h with 20 mg_protein_ g_cellulose_^-1^ of *Tr*Cel7A, *Hi*Cel45A, GH10Xyn, or GH11Xyn. Fucose was used as internal standard.


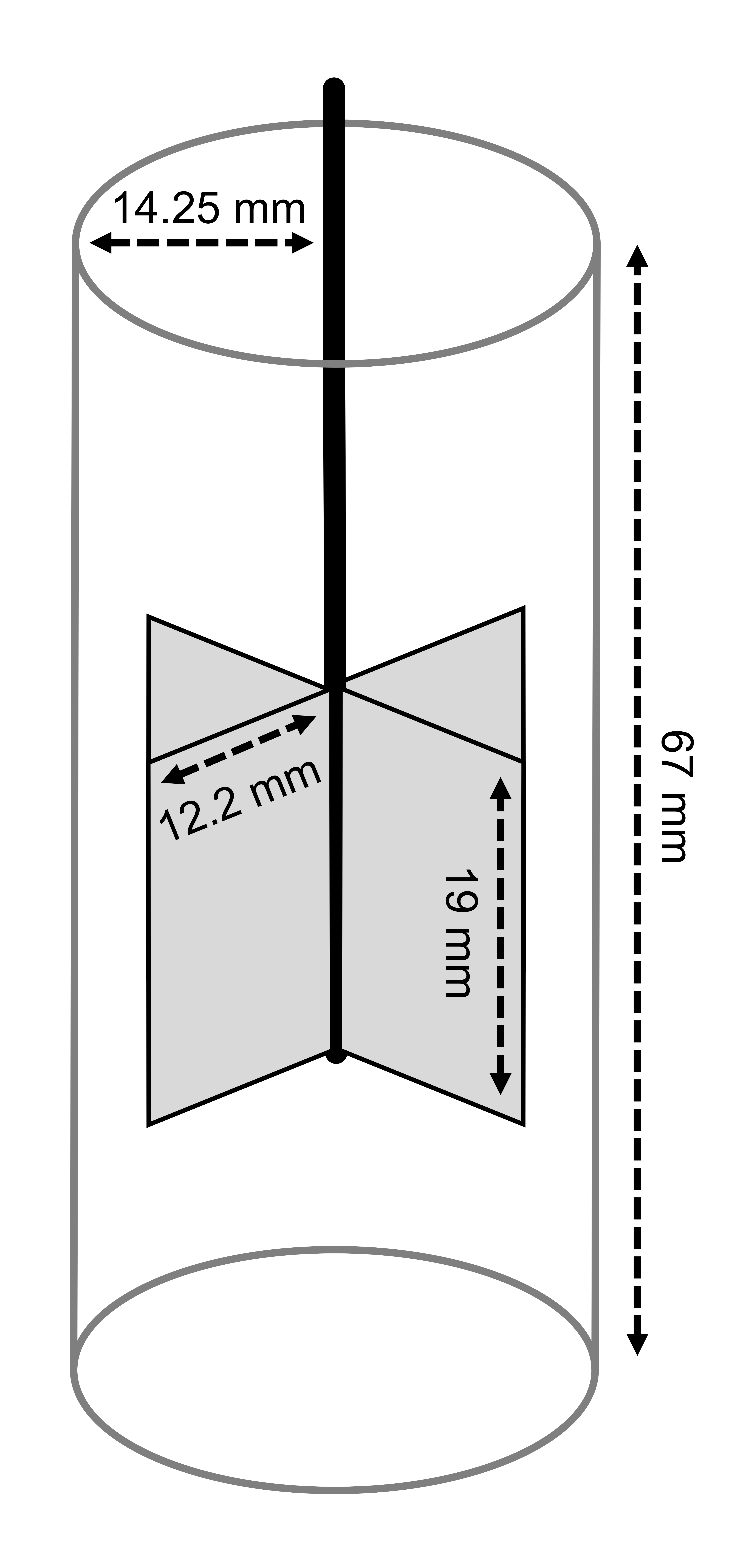


**Fig. S6.** Illustration of the four-bladed vane-in-cup rheometry setup used in this study.


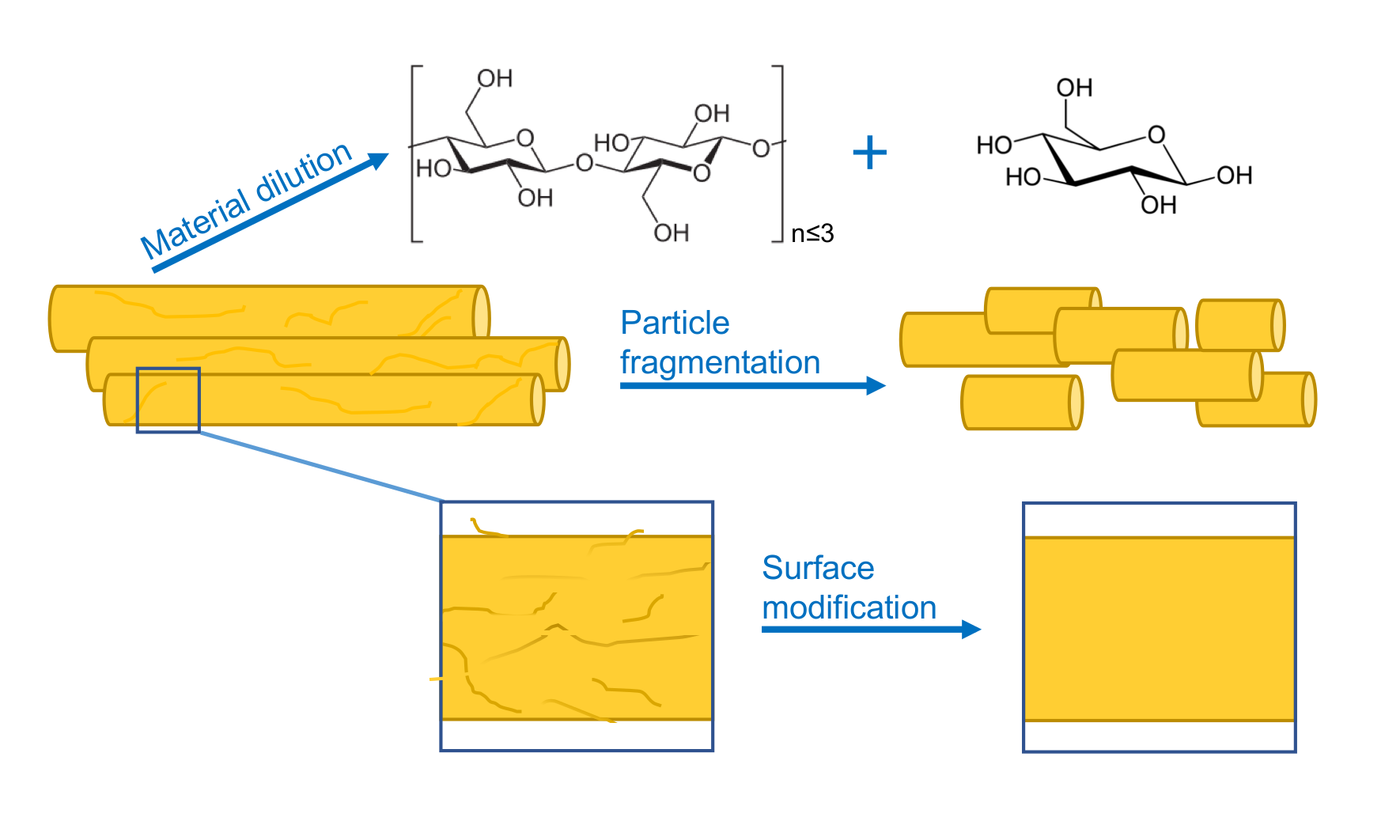


**Fig. S7**. Illustration of the potential enzymatic liquefaction mechanisms of material dilution, particle fragmentation and surface modification/alteration of interparticle interactions.
